# Localization and Registration of 2D Histological Mouse Brain Images in 3D Atlas Space

**DOI:** 10.1007/s12021-023-09632-8

**Published:** 2023-06-26

**Authors:** Maryam Sadeghi, Arnau Ramos-Prats, Pedro Neto, Federico Castaldi, Devin Crowley, Pawel Matulewicz, Enrica Paradiso, Wolfgang Freysinger, Francesco Ferraguti, Georg Goebel

**Affiliations:** 1 Department of Medical Statistics and Informatics, Medical University of Innsbruck, Innsbruck, Austria; 2 Department of Pharmacology, Medical University of Innsbruck, Innsbruck, Austria; 3 Faculty of Engineering, University of Porto, Porto, Portugal; 4 Biomedical Engineering, Johns Hopkins University, Baltimore, United States; 5 KNAW, Netherlands Institute for Neuroscience, Amsterdam, Netherlands; 6 Univ. ENT Hospital, Medical University of Innsbruck, Innsbruck, Austria

**Keywords:** Image registration, 2D in 3D localization, Deep learning, Mouse Brain Mapping

## Abstract

To accurately explore the anatomical organization of neural circuits in the brain, it is crucial to map the experimental brain data onto a standardized system of coordinates. Studying 2D histological mouse brain slices remains the standard procedure in many laboratories. Mapping these 2D brain slices is challenging; due to deformations, artifacts, and tilted angles introduced during the standard preparation and slicing process. In addition, analysis of experimental mouse brain slices can be highly dependent on the level of expertise of the human operator. Here we propose a computational tool for Accurate Mouse Brain Image Analysis (AMBIA), to map 2D mouse brain slices on the 3D brain model with minimal human intervention. AMBIA has a modular design that comprises a localization module and a registration module. The localization module is a deep learning-based pipeline that localizes a single 2D slice in the 3D Allen Brain Atlas and generates a corresponding atlas plane. The registration module is built upon the Ardent python package that performs deformable 2D registration between the brain slice to its corresponding atlas. By comparing AMBIA’s performance in localization and registration to human ratings, we demonstrate that it performs at a human expert level. AMBIA provides an intuitive and highly efficient way for accurate registration of experimental 2D mouse brain images to 3D digital mouse brain atlas. Our tool provides a graphical user interface and it is designed to be used by researchers with minimal programming knowledge.

**Supplementary Information:**

The online version contains supplementary material available at 10.1007/s12021-023-09632-8.

## Introduction

Accurate quantitative and comparative analysis of the anatomical organization of neural circuits of the mouse brain at single-cell resolution is key to elucidate brain functions (Paşca, 2018). In order to standardize and globalize studies across subjects and different modalities, it is crucial to map the experimental brain data to a common coordinate space (Lein et al., 2007; Oh et al., 2014; Papp et al., 2014).

**Fig. 1:**
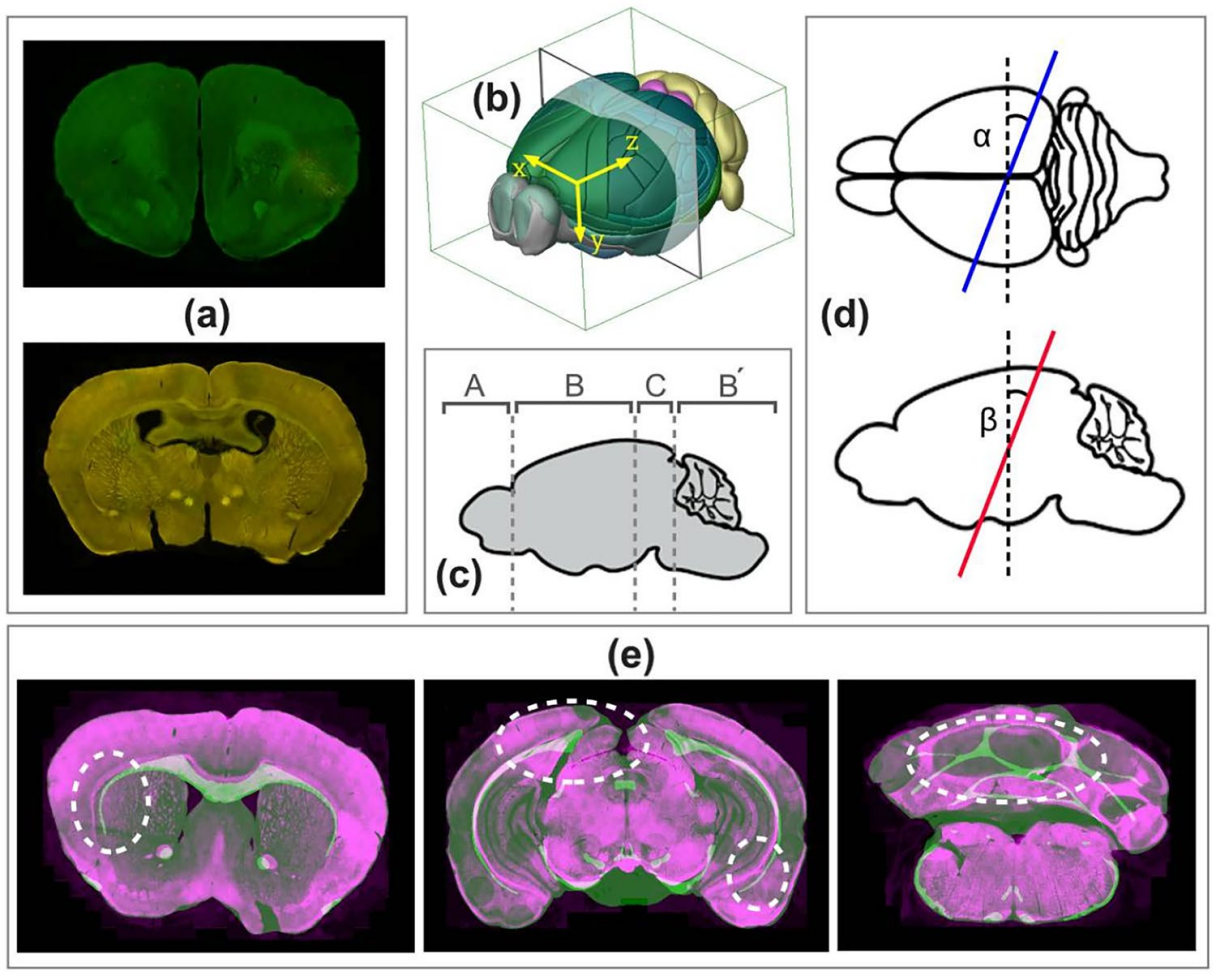
**a** Examples of histological MBIs sliced at non-standard angles. **b** Demonstration of the axes in the 3D mouse brain space: x-axis = left-to-right, y-axis = dorsal-to-ventral and z-axis = anterior-to-posterior, and the origin of the coordinate system. **c** Categorization of the MBIs based on the position along the z-axis **d** Definition of the mediolateral angle ($\alpha$) and the dorsoventral angle ($\beta$) in the horizontal and sagittal views, respectively. **e** Examples of an MBI (purple) superimposed on the registered atlas (green) using Ardent registration showing mismatch of the internal structures (dashed white markings)

Existing mapping methods and tools fall under two major categories: (I) mapping 2D histological mouse brain slice images (MBI) to standard 2D coronal/sagittal atlases (Pallast et al., 2019; Iqbal et al., 2019; Abdelmoula et al., 2014; Piluso et al., 2021; Wang et al., 2022; Majka & Wójcik, 2016) or (II) partial or whole 3D reconstruction of a series of MBIs and then performing 3D-3D registration (Ni et al., 2020; Kim et al., 2015; Wang et al., 2021; Niedworok et al., 2016; Renier et al., 2016; Qu et al., 2022; Jin et al., 2022). With approach (I), prior to the registration step, it is necessary to specify the correct atlas plane matching the histological MBI. Despite the progress in this field, this pre-step is still done manually by the human expert based on visual similarity. This task is challenging and often inaccurately performed since brain slices often have non-standard slicing angles (Fig. 1a). A slightly tilted brain slice can have substantially different anatomical regions compared with the standard axis reference atlas, which is often neglected during conventional analysis. Moreover, localizing the MBIs along the brain anteroposterior (AP) axis is highly dependent on the professional knowledge of the operator, which can introduce a significant bias (Tappan et al., 2019). The drawback of approach (II) is that 3D reconstruction and registration methods are computationally expensive and require a set of consecutive MBIs, which are not always available. In practice, acquired brain datasets are often incomplete due to the researcher’s particular interest in specific brain regions, limited resources, or artifacts during the brain slicing process.

Considering the drawbacks of the aforementioned approaches, a more suitable and efficient solution is the localization of 2D MBIs in the 3D atlas space (Fig. 1b). 2D to 3D localization allows the acquisition of custom atlas planes that match the slice plane orientation and provide superior anatomical precision. The angles to consider when calculating the orientation of the brain slice are the mediolateral angle around the dorsal-to-ventral axis and the dorsoventral angle along the left-to-right axis, $\alpha$ and $\beta$, respectively (Fig. 1d). Several studies have addressed the localization of 2D MBIs in the 3D reference space (Puchades et al., 2019; Xiong et al., 2018; Tappan et al., 2019; Song et al., 2020; Agarwal et al., 2017). Some studies consider the slicing angle and allow tilted atlas extraction, but the angle selection is done manually (Puchades et al., 2019; Agarwal et al., 2017). Other studies use an automatic approach (Xiong et al., 2018; Song et al., 2020), but the iterative nature of the methods is computationally expensive and slow. One study used a deep learning (DL) model to predict the coordinates of the MBIs in the reference space but assumed a uniform slicing angle for all slices (Carey et al., 2022).

**Fig. 2:**
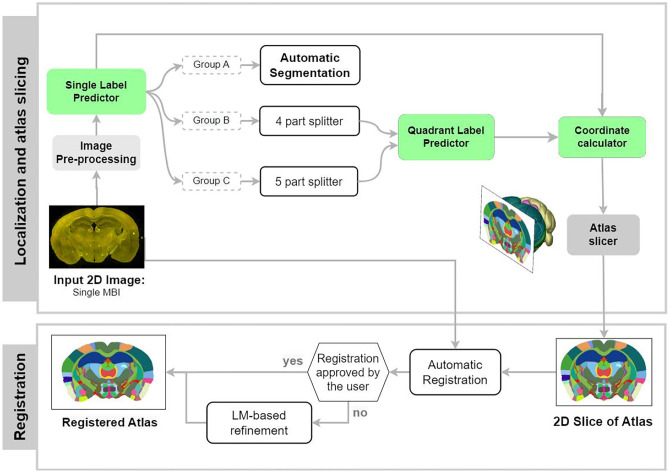
Diagram showing key steps in the workflow of AMBIA’s localization and registration modules. The localization module calculates the AP position d and slicing angles $\alpha$, $\beta$ of the input histological MBI, based on the single label value and the quadrant/quintant label values. It consecutively extracts a 2D atlas by virtually slicing the 3D atlas with the calculated plane coordinates [$\alpha$, $\beta$, d]. The registration module then registers the 2D atlas to the MBI using an automatic deformable registration followed by optional manual landmark (LM)-based refinement by the user

After the atlas plane is determined, it is necessary to perform 2D registration between the MBI and the obtained atlas. Image registration methods are extensively studied in medical images (Maintz & Viergever, 1998) and brain science (Niedworok et al., 2016). Some computational tools have been developed to automate the 2D-2D MBI registration using deformable registration algorithms (Abdelmoula et al., 2014; Xiong et al., 2018; Tappan et al., 2019; Song et al., 2020; Agarwal et al. 2017). Other studies have attempted to automate it using deep learning approaches (Krepl et al., 2021). Although the automatic approaches show promising results, they do not always perform well when histological images contain major artifacts. Furthermore, they require advanced programming knowledge to be used in practice.

Ardent (Neurodata, 2023; Tward et al., 2019) is an open-source registration module based on large deformation diffeomorphic metric mapping (LDDMM) (Beg et al., 2005), outperforming other methods for mouse brain image registration (Bai et al., 2012). Ardent has advanced features including artifact detection and cross-modal support for registering images with different types of contrast. However, although it performs well with outer contours, it fails to consistently register finer inner structures accurately, without manual hyperparameter tuning (Fig. 1e).

In this paper, we present AMBIA (Accurate Mouse Brain Image Analysis), a computational tool for localization and registration of 2D histological MBI in the 3D reference space. AMBIA has a modular design and consists of a localization and a registration module. The localization module is an automated DL-based pipeline that finds the position and orientation of the 2D histological MBI in the 3D brain model and generates a precise matching 2D atlas plane sliced from the 3D atlas.

In the AMBIA registration module, we extend the functionality of the Ardent package to improve the registration on inner structures. We demonstrate the advantage of our method, by comparing the results of fully automatic registration and our hybrid registration method. Moreover, we validate the accuracy of this hybrid registration method, by comparing the results to fully manual registration by human experts as ground truth.

This paper presents a computational tool for the localization and registration of 2D MBIs in a 3D reference atlas space with minimal human intervention. Furthermore, the tool has an intuitive graphical user interface (GUI), to ease the use in practice.

## Materials and Methods

AMBIA mainly consists of two modules; the localization module and the registration module (Fig. 2). The first module consists of an automated DL-based pipeline that finds the position and orientation of the 2D histological MBI, including slicing angles and location in the AP axis of the brain. The module then generates a matching atlas plane by virtually slicing the 3D atlas. The registration module then registers the generated atlas to the input 2D MBI. The details of the data and methods used in the localization and the registration modules are described in the following sections.

### Data and Resources

All animal procedures were performed according to institutional guidelines and were approved by the Austrian Animal Experimentation Ethics Board (animal license numbers 2020$-$0.602.380, BMWFW$-$66.011/0123-WF/V/3b/2017) and in compliance with the European convention for the protection of vertebrate animals used for experimental and other scientific purposes, Animal Experiments Act 2012 (TVG 2012) and the EU Directive 2010/63/EU.

Two different types of datasets were used for training, validation, and evaluation in this study. First type are the histological MBIs scanned in-house using a digital slide scanner (Pannoramic 250, 3DHISTECH ltd, Budapest, Hungary) (fluorescent mode, 20x magnification, 0.2 $\mu$m/pixel). Mouse sections were prepared according to previously published procedures (Ramos-Prats et al., 2022). Briefly, mouse brains were fixed with 4% paraformaldehyde, 15% picric acid in 0.1 M phosphate-buffer (PB), pH 7.2$-$7.4. Slices were cut with a vibratome (Leica Microsystems VT1000S, Vienna, Austria) at a thickness of 50 $\mu$m. Some of the sections were processed for immunofluorescence and then mounted onto gelatin-coated slides and coverslipped with Vectashield (Vector Laboratories) or ProLong Diamond (Thermo Fisher Scientific).

Second type are the serial two-photon tomography (STPT) (Ragan et al., 2012) MBI acquired from transgenic line datasets available at Allen Institute’s Online Public Resource. Please see Table A1 in the Online Resource 1 for a complete list of the transgenic lines used.

In this study, two reference atlases were utilized. First, the Allen Reference Atlas (ARA), is a standard set of 2D coronal slices derived from the Allen Institute’s original adult mouse brain atlas, version 2 (2011) (Allen Institute). Second, the Allen Common Coordinate Framework atlas (CCFv3) (Wang et al., 2020), is a 3D atlas employed for the development of our atlas slicer algorithm and will be referred to as the ACCF. Both atlases are annotated digital resources that delineate and color-code anatomical brain regions, complemented by a systematic and hierarchically organized taxonomy of mouse brain structures.

ARA consists of 132 coronal sections evenly spaced at 100 $\mu$m intervals and annotated to detail for numerous brain structures. The 3D ACCF is available at four resolution levels (100 $\mu$m, 50 $\mu$m, 25 $\mu$m, and 10 $\mu$m) in the Allen institute API (Allen Institute). Here we used the 3D ACCF at 10 $\mu$m resolution.

**Fig. 3:**
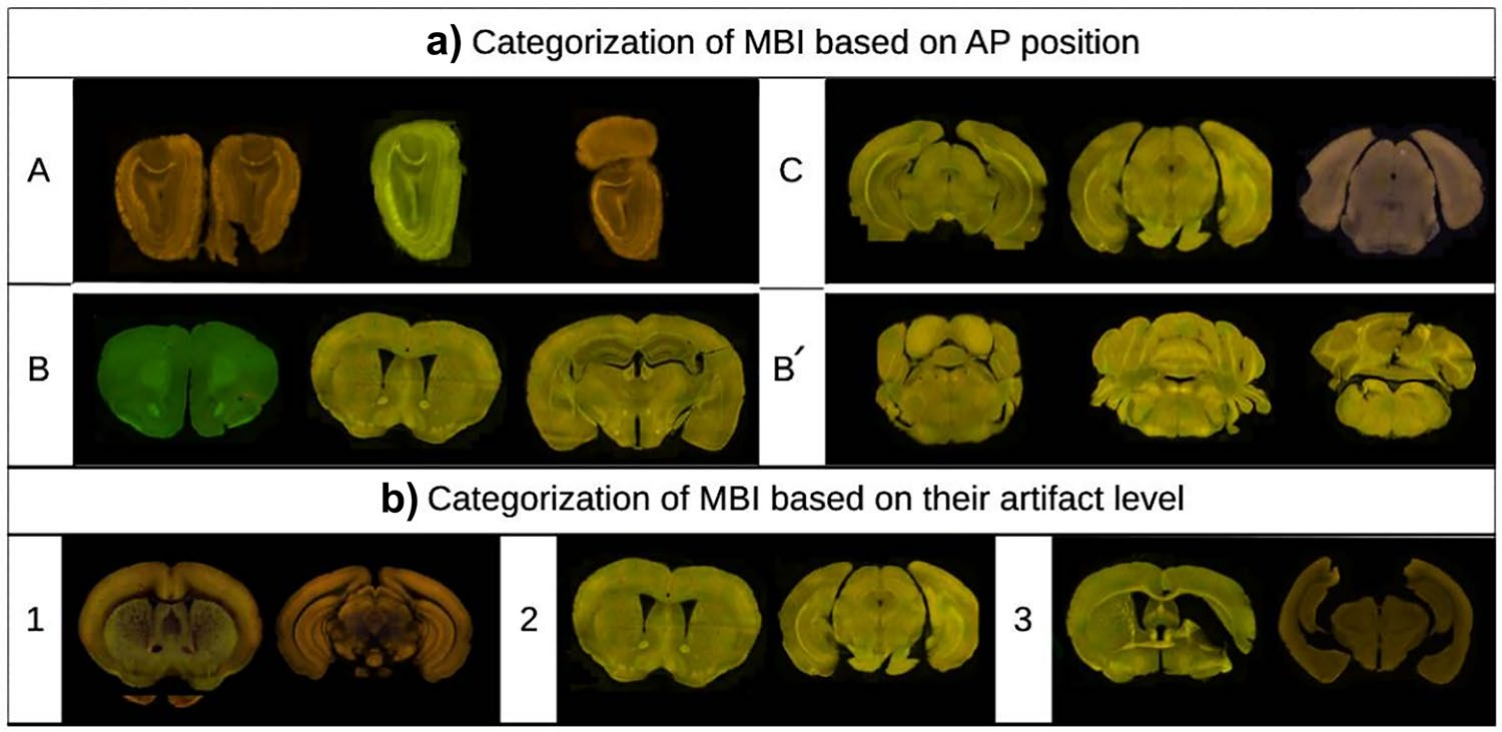
**a** Examples of dataset used for training the localization module, categorized based on the position of MBIs along the AP axis into groups A, B, C and B’ images **b** Examples of MBI used for testing the registration module, categorized based on artifact levels into level 1, 2 and 3

### Localization Module and Atlas Generation

The AMBIA localization module predicts the exact position d and the plane angles [$\alpha$, $\beta$] of the input MBI in the 3D ACCF space. We consider d to be the distance from the origin (Fig. 1b) to the geometric center of the atlas image along the AP axis of the brain. Because of anatomical structural differences occurring in the MBIs across the AP axis, for our approach, we categorized the MBIs into three different groups. The categories based on the ARA numbering system are as follows: group A: 1-22, group B and B’: 23-83 and 104-132, and group C: 84-103 (Fig. 1c). Examples of MBIs for each group are shown in Fig. 3a.

Figure 2 illustrates the localization module pipeline. The Single Label (SL) predictor is a convolutional neural network (CNN) regression model that inputs the 2D MBI and detects approximately the position of the slicing plane along the AP axis without considering the slicing angle. For this purpose, we trained a ResNet50V2 (He et al., 2016) architecture on 4778 images of MBIs, 20% of which were used as a validation set. All the MBIs were manually labeled by three experts with the corresponding Allen atlas section number. The loss function used in the regression model was the Mean Absolute Error (MAE), where a prediction is more penalized the further away it is from the actual ground truth.

As a measure of performance for the SL predictor, we used group accuracy, which computes the accuracy of correct group attribution for each image.

**Fig. 4:**
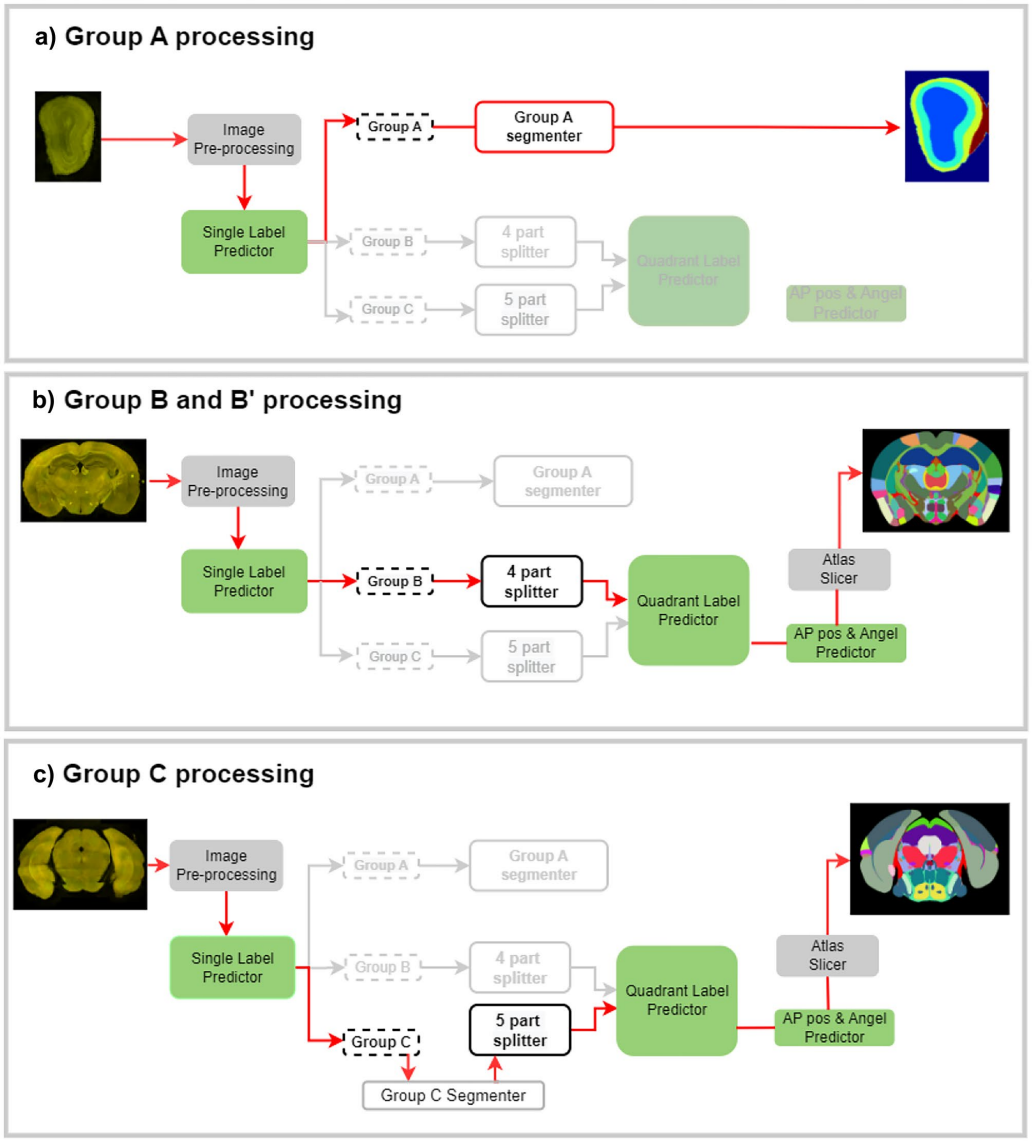
Processing workflow of the localization module for different MBI groups. **a** Group A images are processed via an automatic segmentation method. The group A segmentation model segments brain regions without the need for an atlas. **b** Group B and B’ images are split into 4 quadrants and then fed into the QL predictor. **c** Group C images are split into 5 quintants using the help of the group C segmentation algorithm, and then fed into the QL predictor

Due to limited anatomical structures in group A MBIs, we decided to use segmentation for this group of images instead of proceeding with atlas assignment (Fig. 4a). A U-Net architecture model (Ronneberger et al., 2015) was trained on 192 grayscale 256x256 pixels MBIs with ARA numbers between 1-22. Two experts annotated the images with the following anatomical regions: Main olfactory bulb outer plexiform layer (MOBopl), Main olfactory bulb granule layer (MOBgr), Main olfactory bulb glomerular layer (MOBgl), Accessory olfactory bulb (AOB), and fiber tracts.

**Fig. 5:**
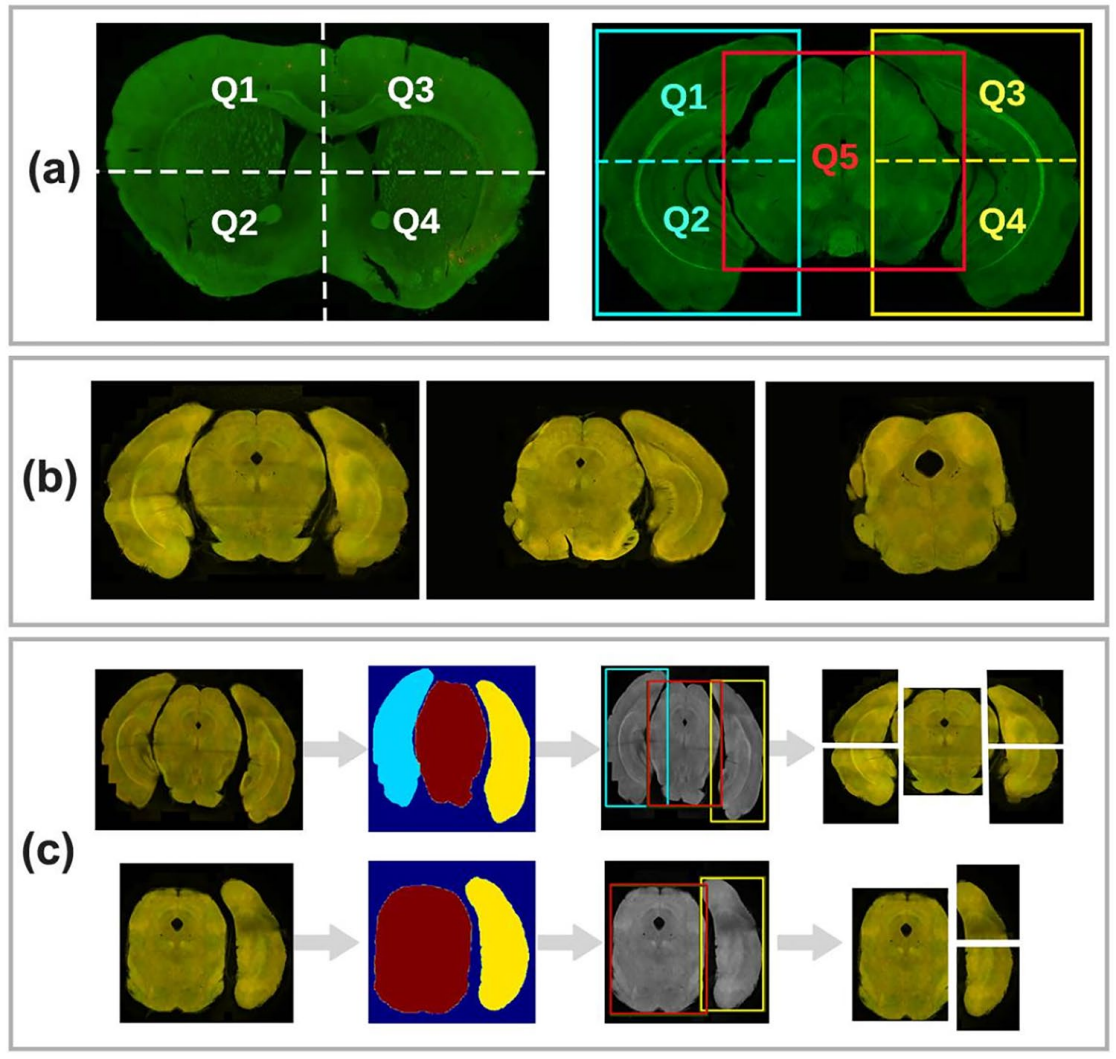
**a** Quadrant splitting and numbering system for group B, B’ (left) and C (right) images, **b** Examples of group C images, which are prone to detachment and losing the left or right cerebrum during processing. **c** Group C segmentation model annotates the regions to help with identifying missing regions, as well as boundaries for cropping five quintants. The images are resized to dimensions of 256x256 pixels and input into the segmentation model, which detects the regions and their respective bounding boxes. Subsequently, the images and bounding boxes are resized back to their original size and aspect ratios

Group B and C images are split into four (“quadrant”) or five (“quintant”) equal segments, respectively, as shown in Fig. 5a. The quadrants are fed separately into the quadrant/quintant label (QL) predictor to associate them to an atlas number referred to as Q labels [Q$_{i}$]$_{i\mathop{=}1:5}$ (Fig. 4b, c). For simplicity, the segments of the five split parts are also referred to as quadrants. The reason for a different splitting strategy between group B and C is due to the fact that sections from the most posterior parts of the brain, ranging 84-103, tend to detach during the slicing process into different components, e.g. left and right cerebrum, and brain stem. This detachment creates two problems. First, the brain stem (Q$_{5}$) is sliced at an entirely different cutting plane as compared to the cerebrum. Therefore, it is necessary to consider that the regions in these MBIs may belong to different ARA planes. Second, it can happen that one or two of these regions are lost in the cutting and staining process (Fig. 5b). Proper identification of these incomplete slices is needed to correctly attribute them to the atlas and perform the registration. The brain stem must be present to consider it an MBI.

**Table 1:** List of abbreviations and full name of brain regions selected to test the registration performance in levels 3, 4 and 5, according to the hierarchical mouse brain structures of the Allen Reference Atlas

**Abbr**	**Name**	**Abbr**	**Name**
**Level 5 brain regions**		VIS	Visual areas
MOB	Main olfactory bulb	PRT	Pretectal region
ORB	Orbital area	CENT	Central amygdalar nucleus
PL	Prelimbic Area	AN	Anterolateral visual area
MO	Somatomotor areas	DEC	Declive
AON	Anterior olfactory nucleus	UVU	Uvula
AI	Agranular insular area	** Level 4 brain regions**	
ACA	Anterior cingulate area	CTXpl	Cortical plate
PIR	Piriform area	CTXsp	Cortical subplate
ILA	Infralimbic area	STR	Striatum
STRv	Striatum ventral region	PAL	Pallidum
STRd	Striatum dorsal region	TH	Thalamus
SS	Somatosensory areas	HY	Hypothalamus
LSX	Lateral septal complex	MBmot	Midbrain, motor related
HIP	Hippocampal region	P	Pons
VISC	Visceral area	MY	Medulla
MTN	Midline group of dorsal thalamus	VERM	Vermal regions
PALd	Pallidum, dorsal region	** Level 3 brain regions**	
PALv	Pallidum, ventral region	CTX	Cerebral cortex
COA	Cortical amygdalar area	CNU	Cerebral nuclei
MED	Medial group of the dorsal thalamus	IB	Interbrain
RSP	Retrosplenial area	MB	Midbrain
LAT	Lateral group of the dorsal thalamus	HB	Hindbrain
AUD	Auditory areas	CBX	Cerebellar cortex
RHP	Retrohippocampal region	CBN	Cerebellar nuclei

We thus next trained a segmentation model to distinguish the brainstem and right and left cerebra to overcome the mentioned problems. The segmentation helps with identifying missing regions, as well as specifying boundaries for cropping five quadrant images (Fig 5c). We trained a U-Net architecture, with a MobileNetV2 (Sandler et al., 2018) base model on 156 MBIs in grayscale, normalized and resized to 256x256 pixels. The QL predictor block consists of a ResNet50V2 CNN regression model trained on 10462 cropped quadrants with their AP position as the label. Labels of the training data were assigned by three experts. The loss function is MAE similar to the one used in the SL classifier. The Q labels for an image were then passed to the next block to calculate the accurate coordinates of the 2D MBI in the 3D ACCF space (in case of group B and B’ images Q$_{5}$ = 0).

For group B and B’ images, a shallow neural network was trained to input the Q labels and output the atlas plane coordinates [$\alpha$, $\beta$, d]. The model was trained on Q values of predictions of our model and angles predicted by the three experts on 250 training data.

**Fig. 6:**
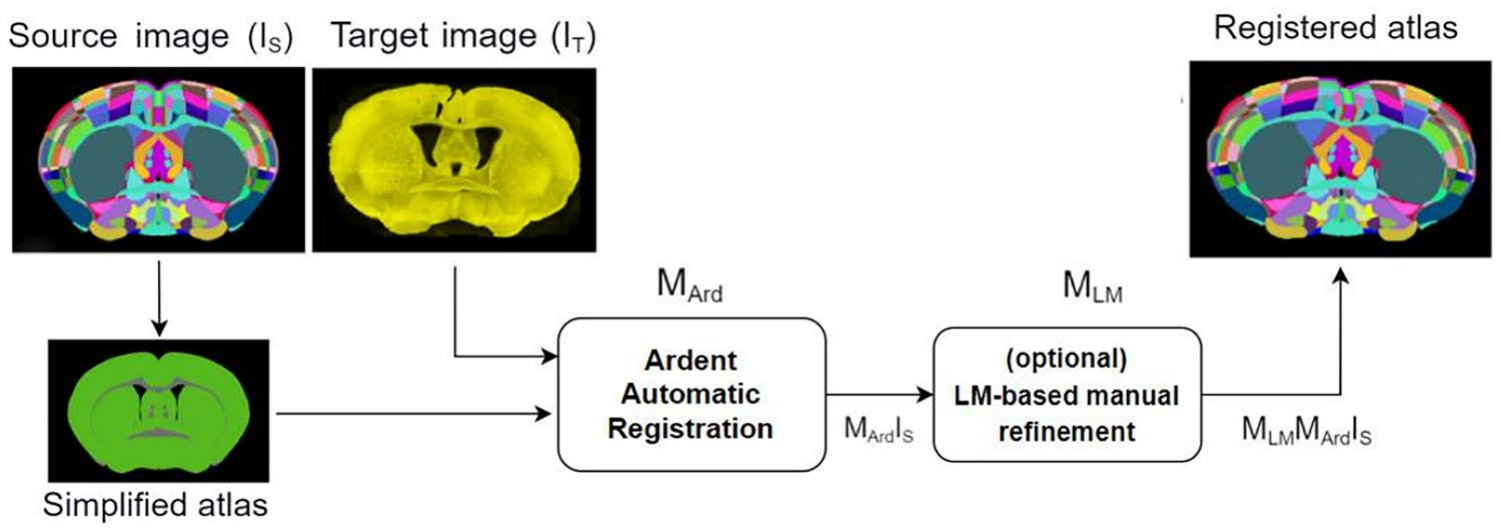
Workflow of AMBIA’s registration module. The module inputs the 2D MBI and 2D atlas image. The atlas image is converted into a simplified representation where fiber tracts are recolored to gray and the rest of the regions are converted to green to create a good contrast similar to visible anatomical structures in an MBI. Ardent registration is performed on the images. The user has the option to refine the registration by choosing landmarks through the GUI

For group C images, a multiplane atlas extraction approach was employed since, due to the occurrence of tissue separation, the atlas was extracted separately for the brain stem, right cerebrum, and left cerebrum in different z-planes. Due to the typical shape of the cerebrum in group C images, in the present study we have considered only the $\beta$ angle. For a more accurate atlas matching, the developed GUI further allows a manual adjustment of the atlas numbers (Q$_{i}$).

### Registration Module

By digitally slicing the ACCF 3D atlas based on the coordinates predicted by our localization pipeline, the 2D atlas corresponding to the input 2D MBI is generated. To map these two images, the following 2D to 2D registration approach was developed.

The AMBIA registration module is built upon the Ardent registration package (Neurodata, 2023) fully written in python and based on the Insight Segmentation and Registration Toolkit (ITK). In our registration approach, shown in Fig. 6, automatic registration is followed by landmark (LM)-based manual refinement. As a first step, Ardent computes a preliminary registration to match the outer boundaries and general anatomical structures of the MBI and atlas. The transformation function M$_{Ard}$ can, therefore, transform the source image (I$_{S}$) to the target image (I$_{T}$), which results in M$_{Ard}$I$_{S}$. Ardent uses mutual information (Maes et al., 1997) as the similarity metric. In order for the similarity measure to perform better, prior to the registration, a simplified representation of the atlas image is generated, because the expert knowledge contained in the anatomical atlas has no counterpart in the visual information of the MBI. In the simplified representation, only fiber tracts are contrasted with gray color against the plain green color background.

The user selects sets of corresponding landmarks (*n* = m, L$_{m}$) in both I$_{T}$ and M$_{Ard}$I$_{S}$ images. The algorithm then automatically selects additional landmarks (*n* = 20, L$_{a}$) along the outer contour of each image to anchor the correct regions and enhance triangulation with more points. The algorithm uses each set of L$_{m}$ + L$_{a}$ landmarks to triangulate each image using the Delaunay’s triangulation algorithm. The corresponding triangles are then registered from the source image to the target image by an affine warp. The transformation function M$_{LM}$ can, therefore, refine the registration of the M$_{Ard}$I$_{S}$ and results in M$_{LM}$M$_{Ard}$I$_{S}$.

The AMBIA GUI provides three registration methods: manual, automated, and semi-automated. In the manual method, users are required to manually select all landmarks, on outer and inner structures, to match the two images. This method is primarily utilized for generating ground truth for evaluating the registration, as detailed in “Evaluation Metrics” section. The automated method relies on the Ardent registration module to register the images. The semi-automated method enables users to manually refine the automatic registration executed by Ardent.

### Implementation

The codes for the presented methods, are implemented in Python 3.7.9.

Localization module: Tensorflow 2.0 backend was used for training to implement the DL-based localization models. The training was performed on a GPU server equipped with a Titan V GPU card, 20 CPU cores, and 256GB of RAM. The prediction of the localization module on one MBI takes 1 s on an intel Core i5 with 8GB RAM.

Registration module: to register one MBI to its corresponding atlas, Ardent takes 30 s, and the LM-based registration takes 20ms after the landmark selection on an Intel Core i5 with 8GB RAM.

### Evaluation Metrics

To evaluate the performance of our localization module, we utilized a test dataset comprising 108 histological MBIs, categorized into three artifact levels (Fig. 3b). Artifact level 1 included 30 images with no artifacts or major morphological changes of the brain, only exhibiting uneven staining, sourced from the Allen Institute’s Transgenic line datasets. Artifact level 2 comprised 44 images with common histological artifacts and morphological changes of the brain, such as tissue shrinkage and uneven staining, scanned in-house. Lastly, artifact level 3 consisted of 34 images with severe histological artifacts, including tears, missing parts, and tissue foldings, also scanned in-house. The images were randomly selected from 10 distinct brains, unseen by the trained model. The test set images were annotated by four experts independently, with single and quadrant labels. The raters assigned a label, a numerical value ranging from 1 to 132 to each whole section, and each quadrant separately.

Due to the interrater variations (four human raters for each group of test images) in labeling the atlas numbers, we have assessed the average label (Avg) and the standard deviation (SD) of their ratings. The accuracy of the module was determined by whether its prediction fell within the range of Avg±2SD referred to as Accuracy 2SD. For fair comparison, each rater is evaluated with the same method against the other three raters.

**Fig. 7:**
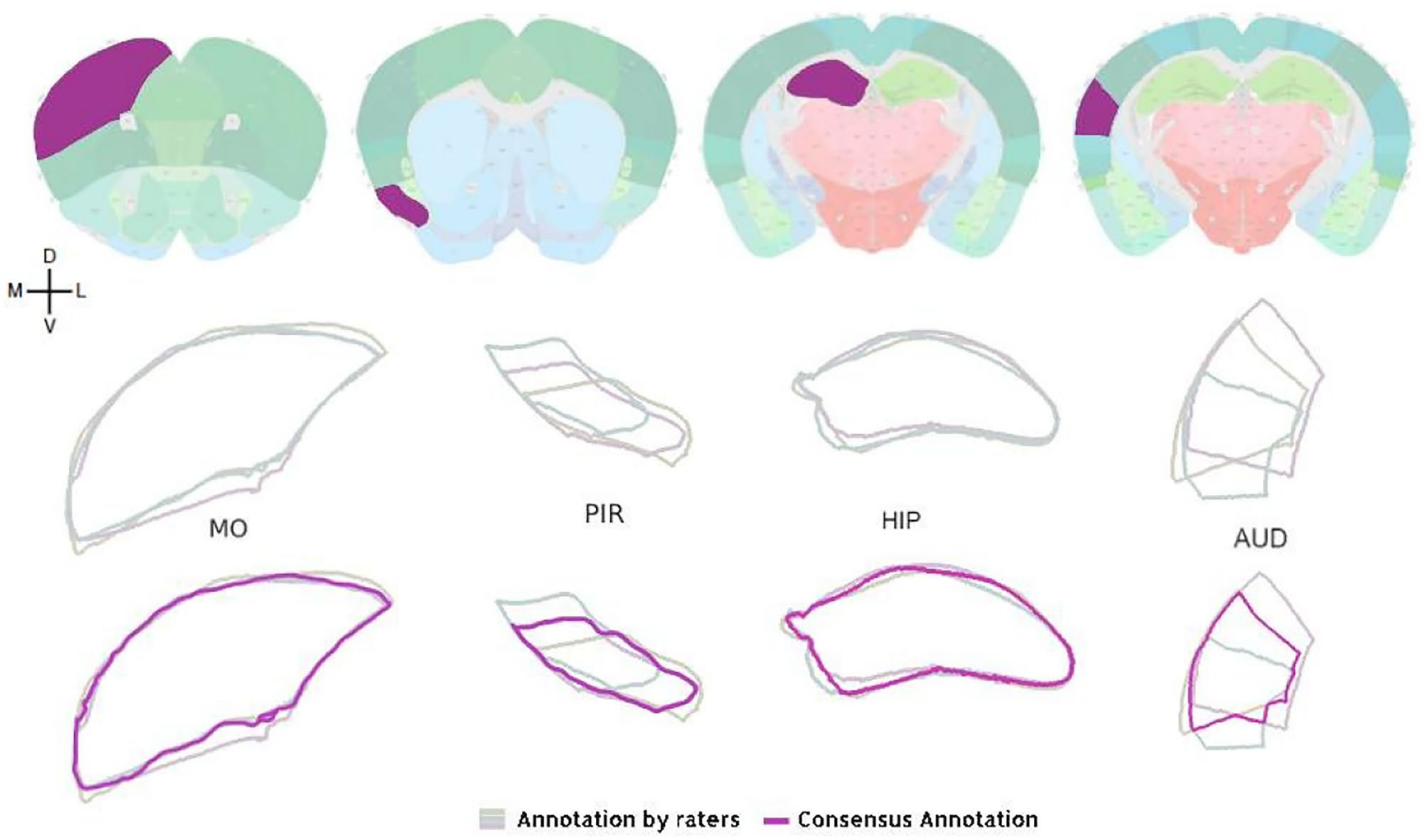
Examples of four anatomical structures out of 30 structures chosen to assess the performance of the registration module. For each brain structure highlighted region on the Allen coronal atlas plane, the segmentation outlines as a result of the registration of the three raters (dim colored lines) the consensus outline for the same structure (bold colored line) determined by the majority vote, are shown

To analyze the performance of our registration module, we chose 60 slices from 3 different brains (*n* = 180) evenly distributed along the AP axis. The images were registered using three different methods (manual, automatic, and semi-automatic) by three experts using AMBIA. The ground truth registration was determined by the consensus of manual registration by the experts and the consensus region was determined using majority vote (Fig. 7). The registration via automatic and semi-automatic methods by each rater was compared to the ground truth registration using the Dice score metric (Dice, 1945), that is commonly used to assess segmentation quality (Leung et al., 2010). The registration of a given rater was also compared to other raters to demonstrate the interrater variability. We performed analyses on multi-level brain structures from the Allen atlas. For a complete list of region names and abbreviations refer to Table 1.

To produce segmentation annotations for the training data and establish ground truth annotations for the evaluation of group A and group C segmentation models, two experts annotated the anatomical regions using an open-source segmentation labeling tool (https://labelbox.com).

The evaluation results were statistically analyzed using the Mann–Whitney U test and Levene’s test, as implemented in the SciPy Python package. A *p-value* threshold of 0.05 was utilized to determine the significance of the differences observed.

It is important to highlight that the variation in the number of experts involved in the evaluation process is not attributed to any specific rationale, but rather stems from the availability of experts at the time the assessments were conducted.

## Results

### Performance of the Localization Module

**Fig. 8:**
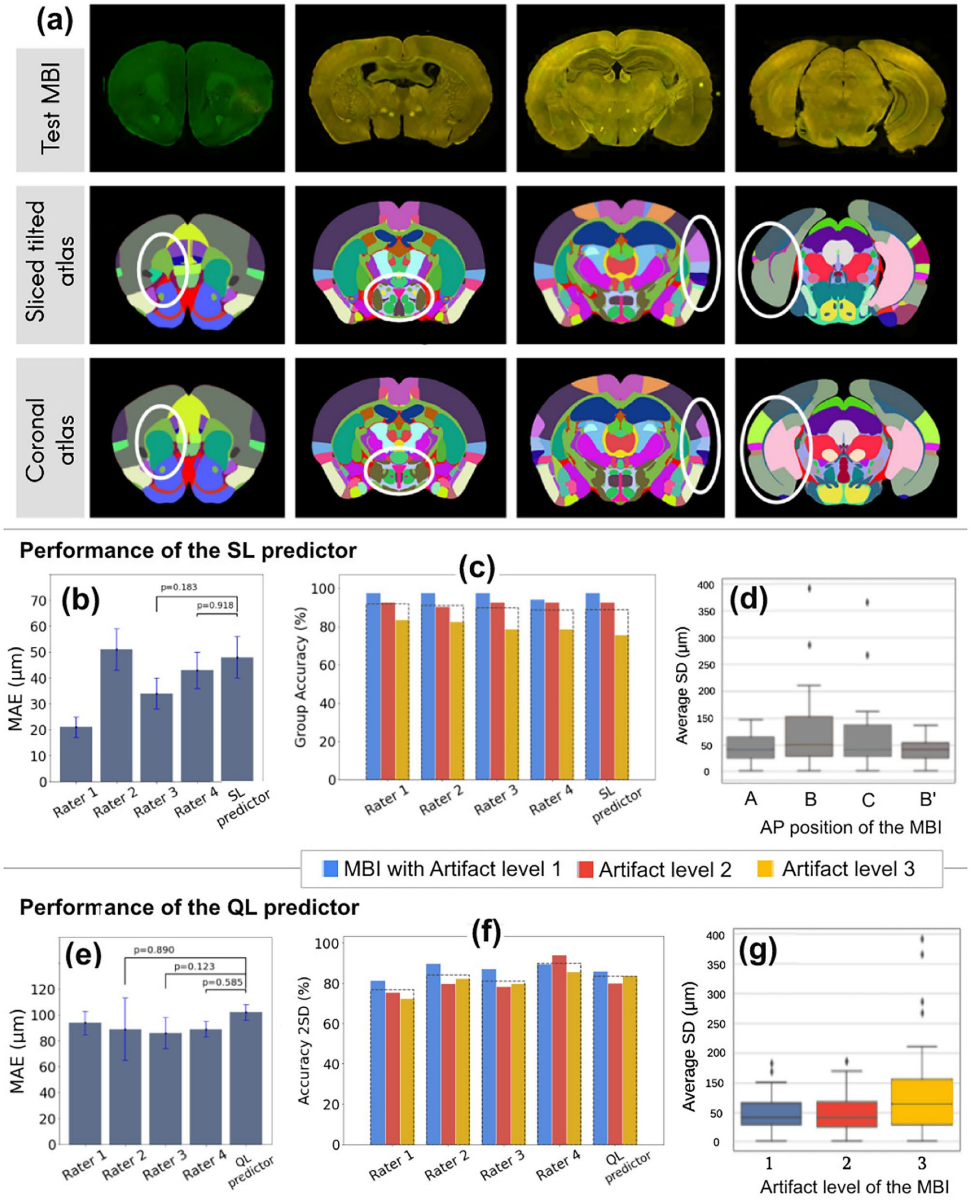
**a** Qualitative and visual evaluation of the performance of the localization module. First row) Histological MBIs from the test set. Second row) the atlas is sliced based on the coordinates and angles predicted by our pipeline. Third row) the atlas is extracted from standard 2D coronal atlas planes without considering the section angles. The white markings highlight the areas where a tilted atlas section has considerable structural difference to the reference coronal atlas plane. **b** MAE of the SL predictor compared with the four raters, which is statistically non-significant to raters 3 and 4. **c** Group Accuracy comparison between four raters and the SL predictor across different artifact groups. The dashed line represents the average group accuracy for all artifact levels. **d** The average standard deviation of the four raters evaluated in different AP positions. **e** MAE of the SL predictor compared with the four raters, which is statistically non-significant to raters 2, 3 and 4. **f** Accuracy 2SD comparison between four raters and the QL predictor across different artifact groups. The dashed line represents the average group accuracy for all artifact levels. **g** Average standard deviation of the four raters evaluated in different artifact levels

Figure 8a demonstrates examples of tilted MBI from the test dataset with their matching sliced atlas image generated by our atlas slicer algorithm based on the coordinates predicted by our pipeline. The standard coronal atlas chosen by the raters is also displayed for comparison. It can be seen that the atlas generated by our pipeline largely matches the tilted MBI, compared to the standard coronal atlas. Some areas are highlighted with white circles to show the difference in regions when considering the slicing angle (Fig. 8a).

The performance of the SL predictor is presented in Fig. 8b–d.The SL predictor achieved an MAE of 48 $\mu$m (Fig. 8b).The SL predictor had no statistically significant difference to raters 3 and 4 (Mann–Whitney U-test, *p-value* = 0.183 and 0.918). Figure 8c shows group accuracy of four raters compared to the SL predictor separated for different artifact groups. The average group accuracy over all artifact levels is displayed with a dashed line. The SL predictor had an average group accuracy of 94.62% for images with artifact level 1 and 2, and 88.8% for all artifact levels (Fig. 8c). It was observed, as shown in Fig. 8d, that the average SD of annotations by human raters were higher in group B and C images as compared to the other two groups.

The performance of the QL predictor is presented in Fig. 8e–g. The QL predictor achieved an average MAE of 102 $\mu$m (Fig. 8e). It had no statistical significant difference to the performance of raters 2, 3 and 4 (Mann–Whitney U-test, *p-value* = 0.890, 0.123 and 0.585). Figure 8f shows Accuracy 2SD of four raters compared to the QL predictor separated for different artifact groups. The average Accuracy 2SD over all artifact levels is displayed with a dashed line. The QL predictor had an average Accuracy 2SD of 86%.

Human raters had an average SD of 87 $\mu$m, 96 $\mu$m, and 137 $\mu$m in labeling images with artifacts of level 1, 2, and 3, respectively (Fig. 8g). This means that the more the sections were distorted or damaged, the higher the inter-rater variability was. We also evaluated the performance of the shallow neural network for the final prediction on the test set. The results showed an MAE of 0.27° and 0.86° for $\alpha$ and $\beta$ angles, respectively.

### Assessment of the Segmentation

The segmentation models for group A and group C were assessed by comparing their segmentation predictions with the expert annotations for their respective test data using the Dice score metric.

**Fig. 9:**
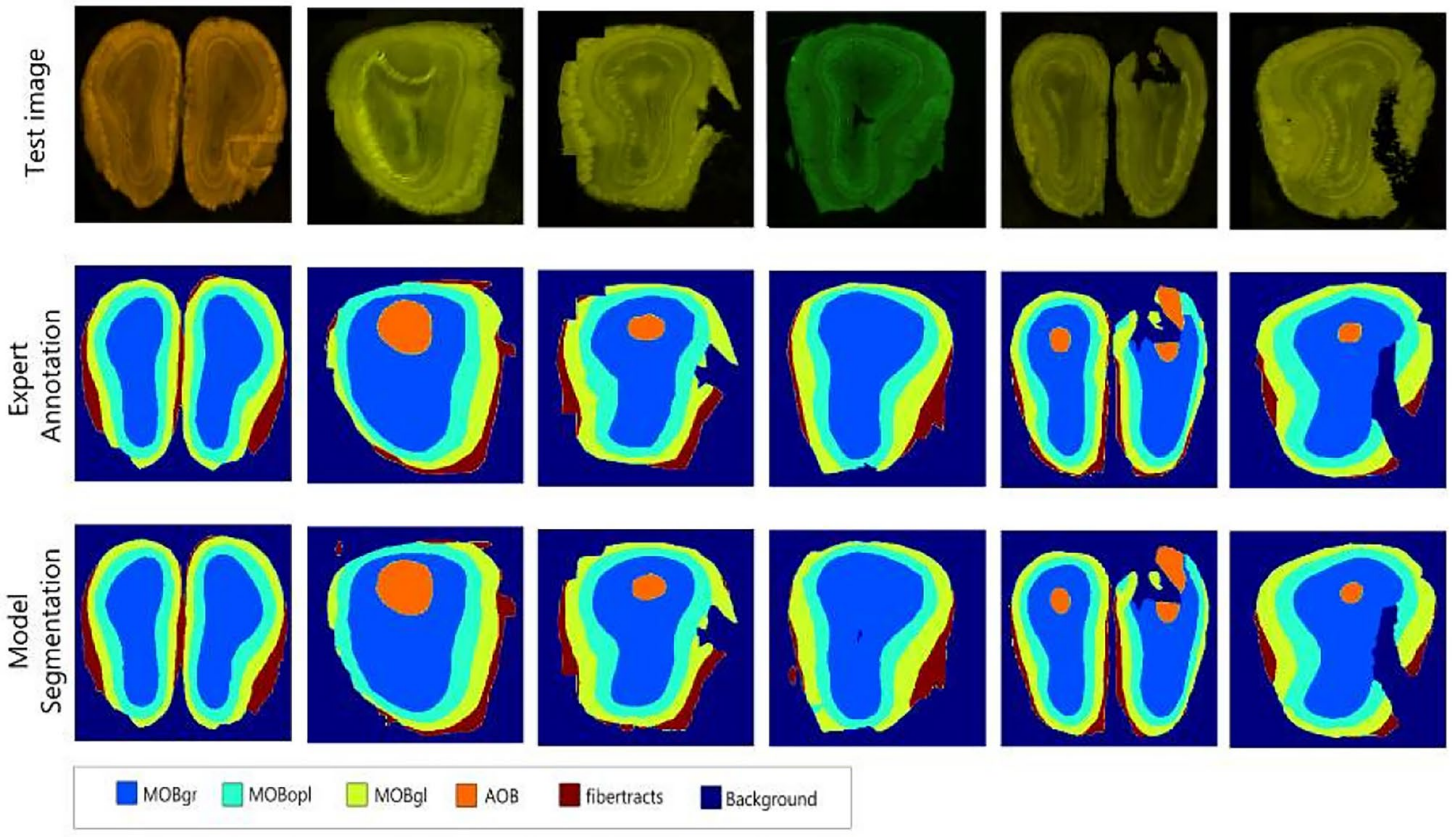
Performance of the group A segmentation model on six test images (first row), expert annotation (second row) compared to the segmentation model performance (third row). Due to limited anatomical structures in group A MBIs, we decided to use segmentation for this group of images. The segmentation model segments five anatomical regions MOBgr, MOBopl, MOBgl, AOB, fibertracts

The group A segmentation model was evaluated on 20 images of group A MBI. The mean Dice score for all regions was 0.924. The Dice scores for separate regions of group A images was 0.92 ± 0.044. Figure 9 shows six images from the test set on the first row, the region annotation labels by an expert, and segmentations by our model in the second and third rows, respectively. It can be seen that the model is able to accurately segment these anatomical brain regions. The group C segmentation model was evaluated on 20 images of group C. It displayed a 100% accuracy in correct region detection and achieved a Dice score of 0.90 and 0.98 for pixel-wise segmentation and bounding box detection, respectively.

### Assessment of the Registration

**Fig. 10:**
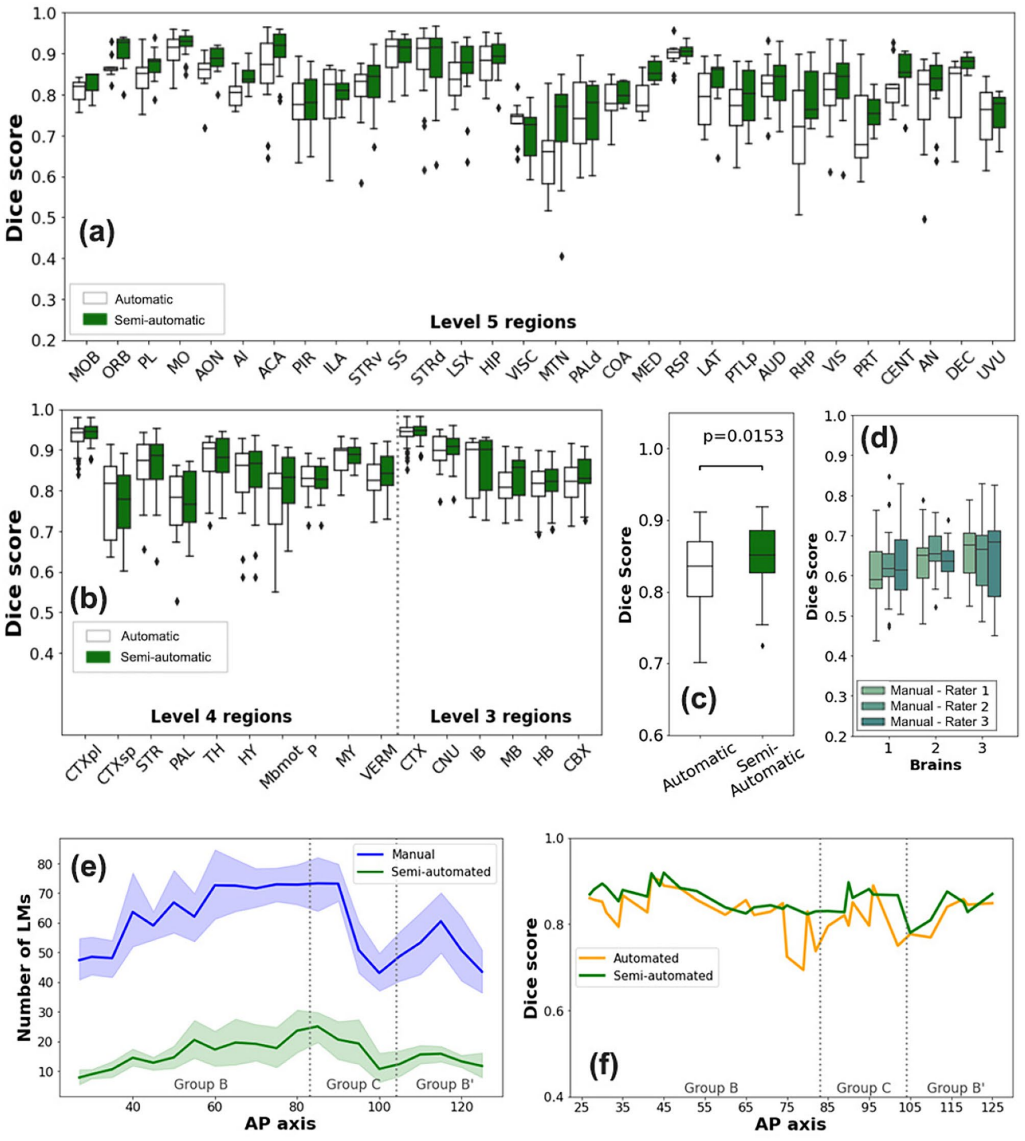
Multi-level assessment of the performance of the AMBIA registration module. **a** Boxplots of dice scores for the automatic (green) and semi-automatic (white) registration methods compared to the ground truth for level 5 brain regions. **b** shows a similar comparison for level 4 and level 3 brain regions. **c** Comparison of manual annotations by three raters for three different test brains. **d** The average (thick line) and range of number of landmarks (shaded area) selected by the four raters for different MBI along the AP axis for the manual and semi-automatic methods. **e** Average dice score for the semi-automatic and automatic registration along the AP axis

The accuracy of the AMBIA registration module was assessed by comparing the results of the registration to a consensus of manual registration considered as the ground truth, based on the comparison of Dice scores for multi-level brain regions. The results of this comparison are shown in the boxplots in Fig. 10a. When pooling the scores of all structures, the average Dice score was 0.86, 0.76, 0.80 for level 3, level 4, and level 5 regions (see Table 1) respectively. Figure 10c shows, when averaged over all regions, the semi-automatic method of AMBIA registration had a significant improvement over the automatic method (average Dice score of 0.80 versus 0.76, Mann–Whitney U-test, *p-value* = 0.0091). In comparison, the manual annotations made by the raters had an average Dice score of 0.71 compared to other raters, indicating a moderate degree of interrater variability (Fig. 10d). It is worth noting that for regions of level 3 and 4, the semi-automatic and automatic methods gave rise to very similar results, but for level 5 regions, the semi-automatic method outperformed the automatic Ardent registration. In addition, for larger regions such as the MO and SS, the difference in performance between the two methods was not as considerable, but in smaller regions such as the MTN and the PRT, the difference was more pronounced (Fig 10a, b). In addition, when comparing the test image groups with varying artifact levels, the semi-automated method exhibited the highest accuracy for the group with an artifact level of 3.

Figure 10e illustrates the number of landmarks that the users selected in the manual and semi-automatic methods, with an average of 60 landmarks chosen in the manual method and an average of 10 landmarks chosen in the semi-automatic method. Manual landmark selection took an average of 120 s, while semi-automatic landmark selection required an average of 40 s. Figure 10f shows that along the AP axis, the semi-automatic method had a more consistent performance than the automatic method (Levene’s test on pooled scores; *p-value* = 0.035).

**Fig. 11:**
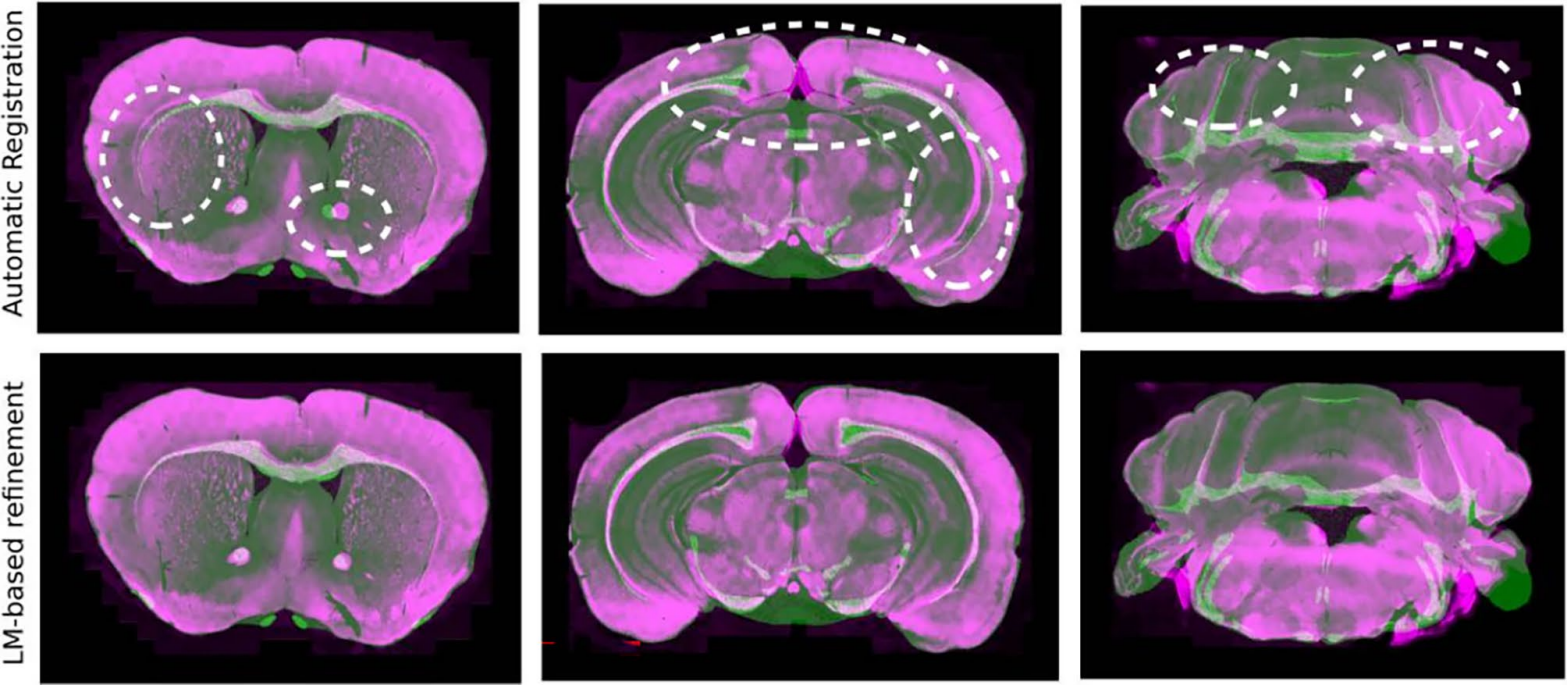
Qualitative evaluation of the registration module. The images show the original MBI, the automatically registered image, and the corrected and refined registration obtained using landmark-based refinement. The MBI (purple) is superimposed on the registered atlas image (green). The figure demonstrates the ability of the registration module to identify and correct misalignment in MBI, as well as the utility of user input in improving the accuracy of the registration. Dashed white markings are used to highlight some regions to show the advantage of manual refinement in the inner structures

Figure 11 demonstrates the ability of the registration module to correct misalignment in MBI, as well as the utility of user input in improving the accuracy of the registration. Dashed white markings are used to highlight some regions to show the advantage of manual refinement in the inner structures.

## Discussion

The recent development of brain-wide neural circuit labeling techniques (Lin et al., 2018; Wang et al., 2019), such as mono-trans-synaptic tracing (Ramos-Prats et al., 2022; Miyamichi et al., 2011) and activity mapping (Roy et al., 2022), as well as the escalation in experiments employing viral transduction of distinct neuronal populations, that require their mapping to precise brain areas, have increased the demand for the development of accurate and automated tools aimed at the anatomical segmentation of individual 2D MBIs. Attempts to map brain anatomical data to an annotated reference atlas depends critically on localization and registration. However, this procedure can be problematic due to variations in brain shape and regions caused by tissue processing, as well as intrinsic biological differences among brains.

To address these issues, we introduce AMBIA, a tool for the localization and registration of 2D histological MBIs with minimal human intervention. The proposed method not only localizes the MBI in the 3D brain along the AP axis, but also calculates the slicing angle, considerably faster compared to 3D reconstruction approaches. A preliminary version of AMBIA has already been used earlier (Ramos-Prats et al., 2022).

By comparing the tilted atlases to the coronal atlases, it can be observed that the precision of the anatomical structure of the annotated atlas can benefit considerably from taking the slicing angle of the slice into account. In addition, the pipeline can assign multi-plane atlases to MBIs with unconnected parts. This is especially beneficial for slices taken from the midbrain, where the brain stem can detach from the cerebrum during the slide preparation process.

Our results suggest that AMBIA’s semi-automatic registration method has comparable accuracy to manual experts, while also offering the great advantage of saving time and effort. Our comparison of semi-automatic and automatic registration methods indicates that the semi-automatic method offers higher accuracy compared to the automatic one, particularly in level 5 regions. When examining the results grouped by artifact level, we found that higher inter-rater variability in images with an artifact level of 3 may account for the higher accuracy of the AMBIA registration module in this group. Additionally, when comparing the outcomes of region groups with different hierarchical levels, it was observed that level 5 regions exhibited a higher Dice score than level 4 regions. This may be partially due to the greater number of smaller regions which tend to have higher inter-rater variability in expert annotations. This variability leads to a consensus region that covers a larger area, subsequently increasing the Dice score. It is appropriate to point out the limitations of our tool, AMBIA, in terms of accuracy and generalizability. One limitation of the localization module is the higher error in detecting the $\beta$ compared to the $\alpha$ angle. The high symmetry in the two brain hemispheres facilitates the identification of the $\alpha$ angle in the left-right axis, whereas the $\beta$ angle is harder to define for human raters because of the lack of clear landmarks in distinct areas in the AP axis. Since the model is trained on human-annotated data, it is similarly biased towards lower accuracy on $\beta$. Second, while the current implementation is tailored specifically to the Allen CCFv3 atlas, it is conceivable that the approach could be modified for use with other atlas frameworks. However, this would require considerable modifications to accommodate different atlas structures and coordinate systems. Despite these limitations, we hope that our work can inspire researchers to adapt our approach for a broader range of atlases and templates, thereby expanding the applicability of the methodology to a wider array of research contexts.

It is worth mentioning that while the present study focuses on histological MBIs scanned with a digital slide scanner, our methods have the potential to be applied to a variety of other modalities and domains. The normalization, downsampling, and grayscale transformation of the images used to train the localization module suggest that it may perform with similar accuracy on MBI scanned using different scanners and stained using various staining procedures. Similarly, the use of a simplified version of an atlas in the registration module suggests that it may be able to register a wide range of datasets that exhibit the anatomical features of brain regions, including MRI images. Future research could explore the validity and generalizability of these methods in these and other contexts.

Finally, one notable feature of AMBIA is its modular design, which allows for the integration of various cell detection methods through the use of a placeholder module in the pipeline. This flexibility enables researchers to employ a range of analyses to suit the needs of their specific study. To facilitate the wider use of AMBIA in practice, we have implemented all of its functionalities and modules in a GUI that is easy to use and requires minimal programming knowledge. Figures A1-6 in the Online Resource 2 present the GUI screenshots representing various stages of the process.

## Information Sharing Statement

Our pipeline is made open access for the scientific community. The source code of AMBIA can be obtained at https://github.com/mrymsadeghi/AMBIA. The original Ardent package can be found at https://github.com/neurodata/ardent. A modified version of Ardent is integrated in the AMBIA code. All AMBIA modules can be executed from its GUI. We also provide a step by step tutorial, test data and a "Best Practices" page within AMBIA's GitHub repository to promote its adoption and optimize performance across diverse experimental contexts.

## Supplementary Information

Below is the link to the electronic supplementary material.

Supplementary file 1 (pdf 52 KB)

Supplementary file 2 (pdf 4982 KB)

## Data Availability

All data is available upon reasonable request. Please contact the corresponding author with requests.

## Code Availability

Our pipeline is made open access for the scientific community. The source code of AMBIA can be obtained at https://github.com/mrymsadeghi/AMBIA. The original Ardent package can be found at https://github.com/neurodata/ardent. A modified version of Ardent is integrated in the AMBIA code. All AMBIA modules can be executed from its GUI. We also provide a step by step tutorial, test data and a "Best Practices" page within AMBIA’s GitHub repository to promote its adoption and optimize performance across diverse experimental contexts.
