# Supplementary material for: Localization and Registration of 2D Histological Mouse Brain Images in 3D Atlas Space
Source: Neuroinformatics. 2023 Jun 26;21(3):615–30. doi: 10.1007/s12021-023-09632-8 (PMC10406728; doi:10.1007/s12021-023-09632-8)
Supplement: Supplementary file 1 — (pdf 52 KB) [file 12021_2023_9632_MOESM1_ESM.pdf]

Journal: Neuroinformatics, Springer

Localization and registration of 2D histological mouse brain images in 3D atlas space

Maryam Sadeghi[1*], Arnau Ramos-Prats[2], Pedro Neto[3], Federico Castaldi[2], Devin Crowley[4], Pawel Matulewicz[2], Enrica Paradiso[5], Wolfgang Freysinger[6], Francesco Ferraguti[2] and Georg Goebel[1]

[1*]Department of Medical Statistics and Informatics, Medical University of Innsbruck, Innsbruck, Austria.
[2]Department of Pharmacology, Medical University of Innsbruck, Innsbruck, Austria.
[3]Faculty of Engineering, University of Porto, Porto, Portugal.
[4]Biomedical Engineering, Johns Hopkins University, Baltimore, United States.
[5]KNAW, Netherlands Institute for Neuroscience, Amsterdam, Netherlands.
[6]Univ. ENT Hospital, Medical University of Innsbruck, Innsbruck, Austria.
[*]Corresponding author(s). E-mail(s): maryam.sadeghi@i-med.ac.at

Online Resource 1

Table A1- List of transgenic line datasets used for the training and test of the SL and QL predictors in the localization module of the AMBIA tool

**Name of the transgenic line**		
Calb1-IRES2-Cre;Ai14	Penk-IRES2-Cre-neo;Ai14	Dbh-Cre_KH212;Ai14
Foxp2-IRES-Cre;Ai14	Slc6a5-Cre_KF109;Ai14	Htr3a-Cre_NO152;Ai14
Ntsr1-Cre_GN220;Ai14	Chrna2-Cre_OE25;Ai14	Tacr1-T2A-Cre;Ai14
Slc17a8-iCre;Ai14	Gpr26-Cre_KO250;Ai14	Dlg3-Cre_KG118;Ai14
Calb1-T2A-dgCre;Ai14	Plxnd1-CreER;Ai14	Kcng4-Cre;Ai14
Gad2-IRES-Cre;Ai14	Sst-IRES-Cre;Ai14	Rorb-IRES2-Cre;Ai14
PB-mCitrine_P038	Crh-IRES-Cre_BL;Ai14	Esr2-IRES2-Cre;Ai14
Slc17a8-IRES2-Cre;Ai14	Grik4-Cre;Ai14	Ndnf-IRES2-dgCre-neo;Ai14
Calb2-IRES-Cre;Ai14	Prkcd-GluCla-CFP-IRES-Cre;	Scnn1a-Tg3-Cre;Ai14
Gal-Cre_KI87;Ai14	Ai14	Trib2-F2A-CreERT2;Ai14
PB-mCitrine_P170	Syt6-Cre_KI148;Ai14	Etv1-CreERT2;Ai140
Slc6a3-Cre;Ai14	Grp-Cre_KH288;Ai14	Nos1-CreERT2;Ai14
Cart-IRES2-Cre;Ai14	Pvalb-IRES-Cre;Ai14	Sim1-Cre_KJ18;Ai14
Glt25d2-Cre_NF107;Ai14	Tac1-IRES2-Cre;Ai14	Vip-IRES-Cre;Ai14
Pdyn-T2A-CreERT2;Ai14	Cux2-CreERT2;Ai14	Fezf2-CreER;Ai14
Slc6a4-Cre_ET33;Ai14	Htr1a-IRES2-Cre;Ai14	Nr5a1-Cre;Ai14
Chat-IRES-Cre-neo;Ai14	Rbp4-Cre_KL100;Ai14	Vipr2-IRES2-Cre;Ai14
Gnb4-IRES2-CreERT2;Ai140	Tac2-IRES2-Cre;Ai14	
