# Supplementary material for: Localization and Registration of 2D Histological Mouse Brain Images in 3D Atlas Space
Source: Neuroinformatics. 2023 Jun 26;21(3):615–30. doi: 10.1007/s12021-023-09632-8 (PMC10406728; doi:10.1007/s12021-023-09632-8)
Supplement: Supplementary file 2 — (pdf 4982 KB) [file 12021_2023_9632_MOESM2_ESM.pdf]

Journal: Neuroinformatics, Springer

Localization and registration of 2D histological mouse brain images in 3D atlas space

Maryam Sadeghi[1*], Arnau Ramos-Prats[2], Pedro Neto[3], Federico Castaldi[2], Devin Crowley[4], Pawel Matulewicz[2], Enrica Paradiso[5], Wolfgang Freysinger[6], Francesco Ferraguti[2] and Georg Goebel[1]

[1*]Department of Medical Statistics and Informatics, Medical University of Innsbruck, Innsbruck, Austria.
[2]Department of Pharmacology, Medical University of Innsbruck, Innsbruck, Austria.
[3]Faculty of Engineering, University of Porto, Porto, Portugal.
[4]Biomedical Engineering, Johns Hopkins University, Baltimore, United States.
[5]KNAW, Netherlands Institute for Neuroscience, Amsterdam, Netherlands.
[6]Univ. ENT Hospital, Medical University of Innsbruck, Innsbruck, Austria.
[*]Corresponding author(s). E-mail(s): maryam.sadeghi@i-med.ac.at

Online Resource 2

The graphical user interface (GUI) of AMBIA

Here we provide screenshots of the graphical user interface (GUI) of AMBIA to analyse 2D histological mouse brain slice images (MBI). To facilitate the wider use of AMBIA in practice, we have implemented all of its functionalities and modules in a GUI that is easy to use and requires minimal programming knowledge. Figures A1-6 presents the GUI screenshots representing various stages of the process. Our pipeline is made open access for the scientific community. The source code of AMBIA can be obtained at https://github.com/mrymsadeghi/AMBIA.

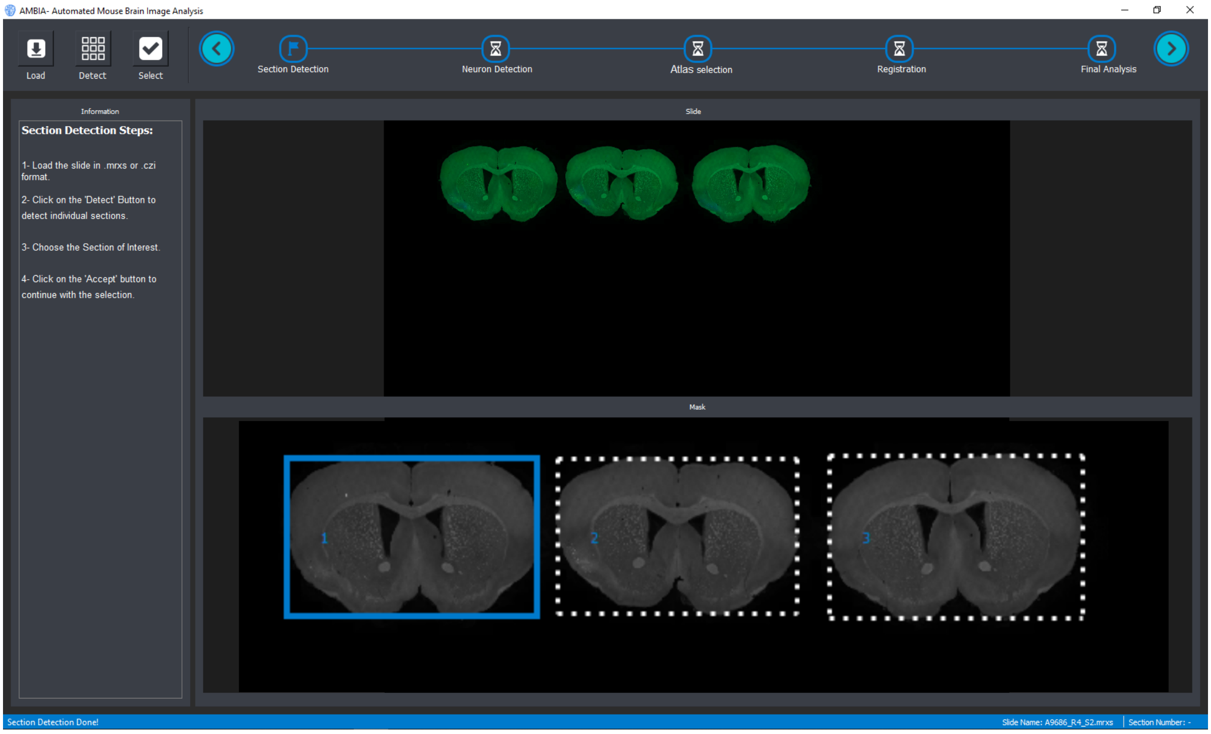

Fig. A1 In the first stage the user can load a whole slide image which may contain multiple MBIs. By clicking the "Detect" button, individual MBIs on the slide image are detected and displayed in the bottom panel. The user can then choose which MBI to proceed with. By clicking the "Accept" button the user is directed to the next stage which is the Neuron detection stage.

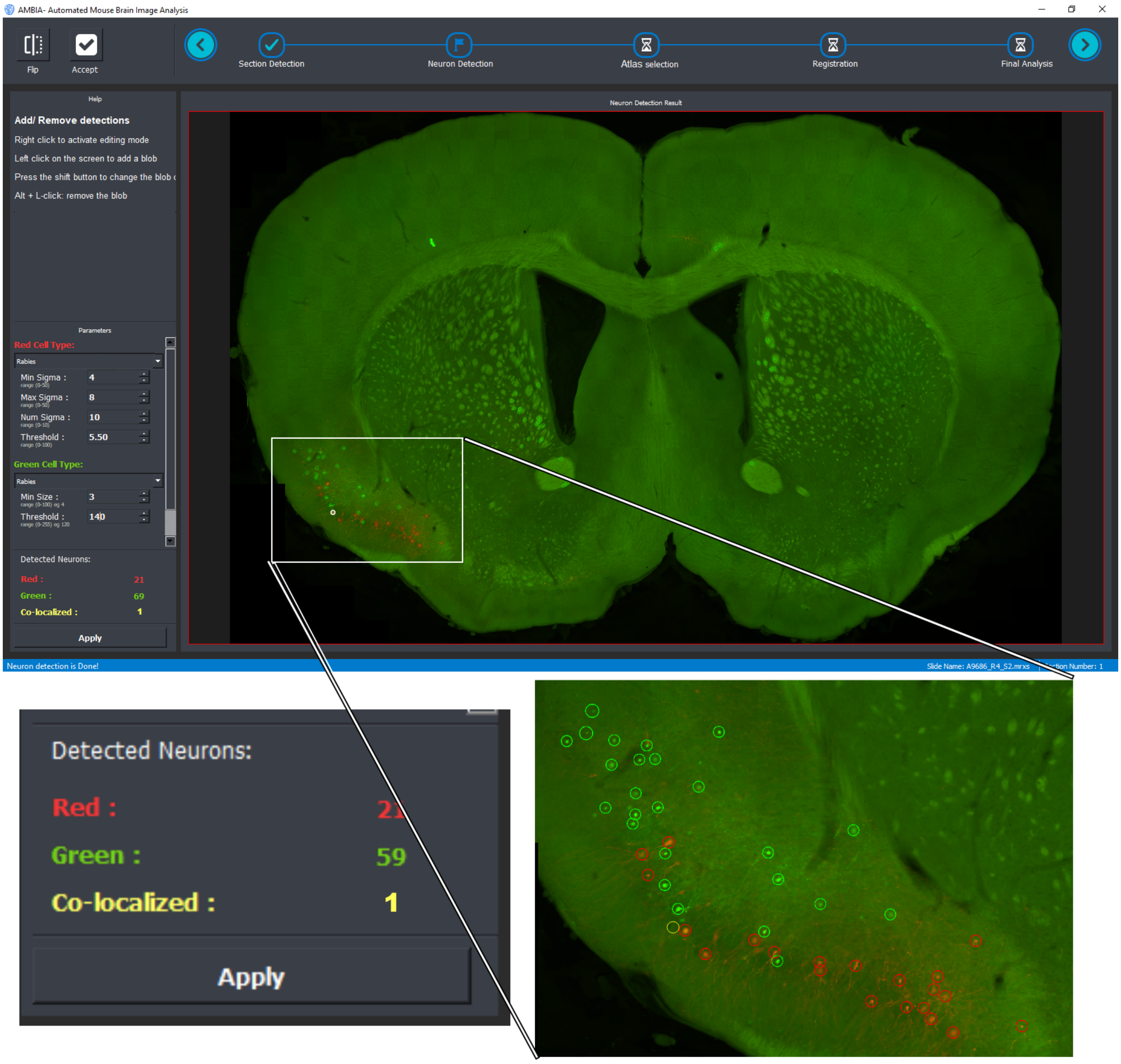

Fig. A2 In the neuron detection stage, the individual selected MBI is displayed. Any customised cell detection python code can be implemented. AMBIA allows for the integration of various cell detection methods through the use of a placeholder module in the pipeline. This flexibility enables researchers to employ a range of analyses to suit the needs of their specific study. In the left panel parameters of the cell detection algorithm can be adjusted and by clicking the “Apply” button, the neurons are detected, counted and displayed on the screen. The user can also add or remove detected neurons by clicking on them on the displayed image. By clicking the “Accept” button the user is directed to the next stage which is the localization stage.

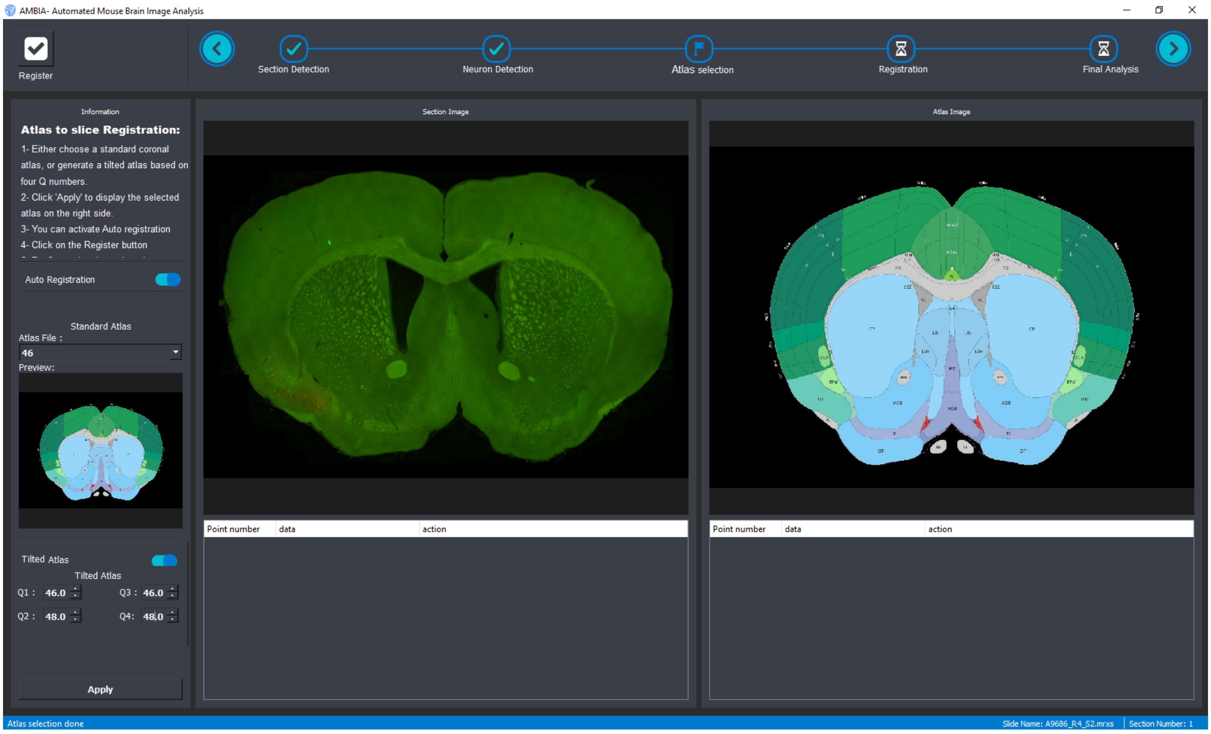

Fig. A3 In the localization stage, the localization module detects the corresponding atlas plane and displays the Allen mouse brain atlas according to the atlas number predicted. The user can choose a different atlas number manually. Additionally by switching the tilted atlas toggle on, the Q values for the quadrants are predicted and displayed and the corresponding tilted atlas is generated by the AMBIA atlas generator algorithm. Here also the user can modify the Q values if necessary. After choosing the atlas plane the user can click the "Accept" button, which performs the automatic registration between the MBI and the selected/generated atlas plane.

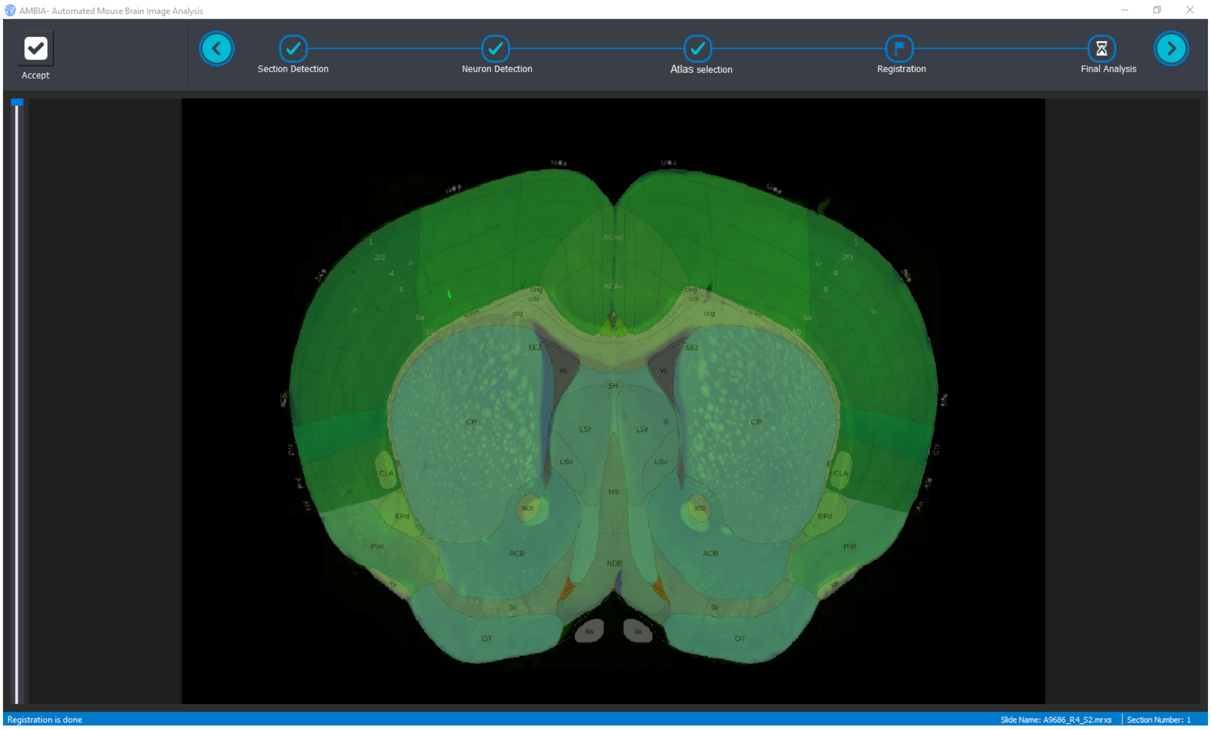

Fig. A4 After the automatic registration is performed an overlaid version of the registered MBI on the atlas is displayed to the user to be able to assess the quality of the automatic registration. A slide bar on the left side of the screen allows the user to change the relative opacity of the two overlaid images.

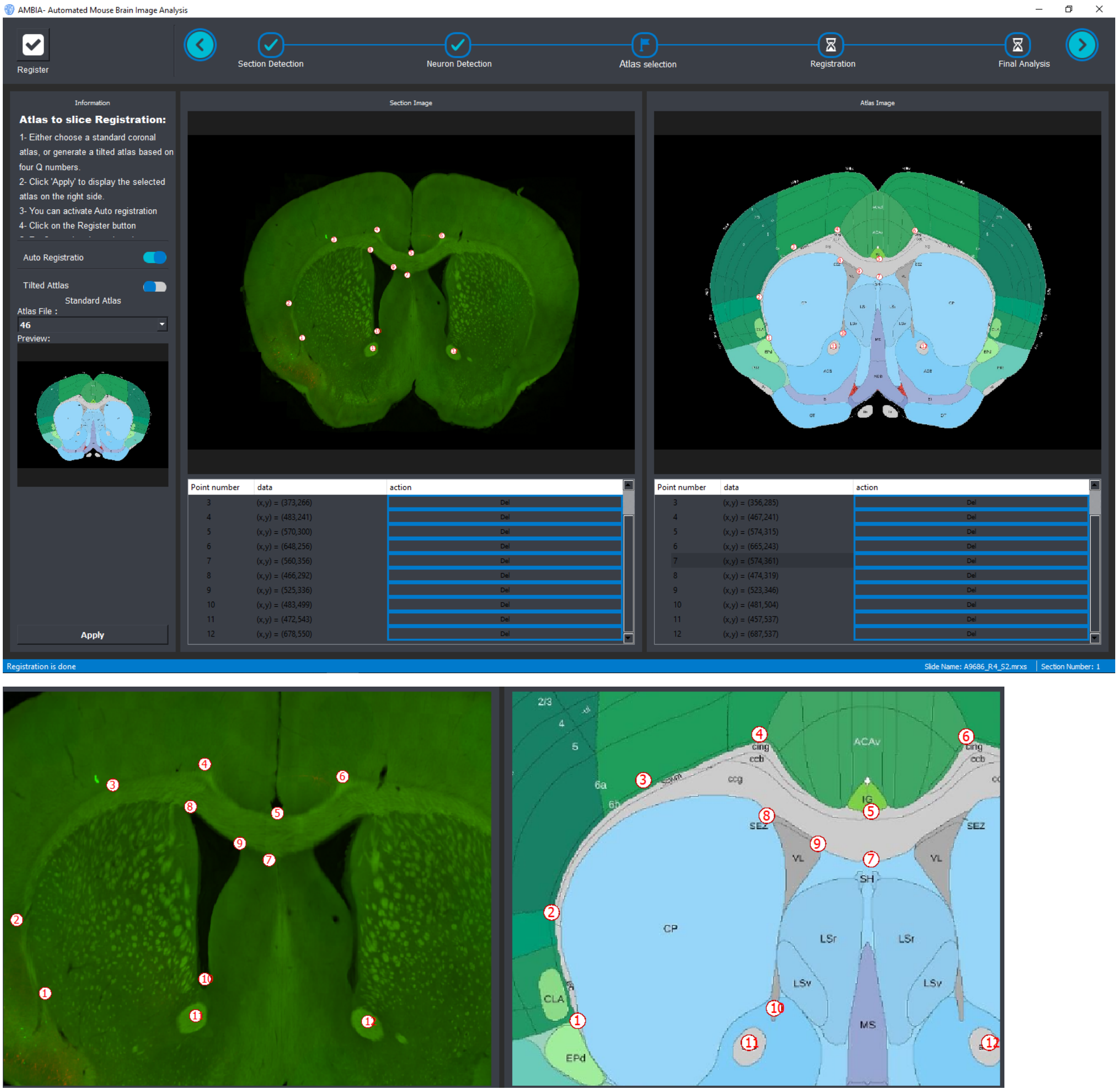

Fig. A5 After examining the automatic registration, the user can go back and refine the registration by manually choosing landmark(LM)s on the MBI and the atlas image. The coordinates of the LMs are displayed in the panels below each image, and can be deleted if necessary. The LM-based registration is confirmed by clicking the “Accept” button, which redirects the user to the registration preview stage to assess the quality of the registration visually. This iterative process can be repeated until the desired level of registration is achieved.

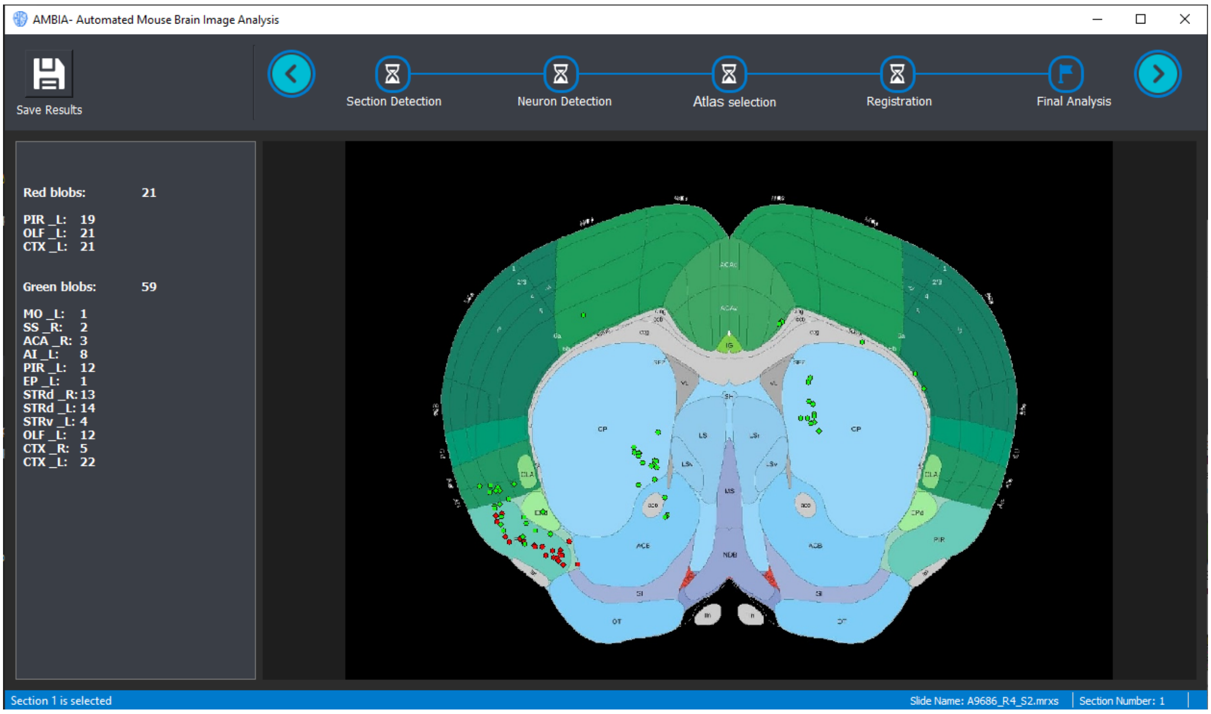

Fig. A6 After confirming the registration preview stage by clicking the “Accept” button, the user is redirected to the final report stage. The coordinates of the detected neurons are transformed into the atlas space using the registration transform functions, and their location is displayed on the atlas. The number of detected neurons in each brain region is displayed in the left panel and can be exported as an Excel sheet and a text file for further analysis.
